# Classification of a moderately oxygen-tolerant isolate from baby faeces as *Bifidobacterium thermophilum*

**DOI:** 10.1186/1471-2180-7-79

**Published:** 2007-08-21

**Authors:** Ueli von Ah, Valeria Mozzetti, Christophe Lacroix, Ehab E Kheadr, Ismaïl Fliss, Leo Meile

**Affiliations:** 1 Institute for Food Science and Nutrition, Laboratory of Food Biotechnology, Swiss Federal Institute of Technology, ETH Zentrum, Zürich, Switzerland; 2 Dairy Research Group STELA, Pavillon Paul Comtois, Université Laval, Québec, Canada

## Abstract

**Background:**

Bifidobacteria are found at varying prevalence in human microbiota and seem to play an important role in the human gastrointestinal tract (GIT). Bifidobacteria are highly adapted to the human GIT which is reflected in the genome sequence of a *Bifidobacterim longum *isolate. The competitiveness against other bacteria is not fully understood yet but may be related to the production of antimicrobial compounds such as bacteriocins. In a previous study, 34 *Bifidobacterium *isolates have been isolated from baby faeces among which six showed proteinaceous antilisterial activity against *Listeria monocytogenes*. In this study, one of these isolates, RBL67, was further identified and characterized.

**Results:**

*Bifidobacterium *isolate RBL67 was classified and characterized using a polyphasic approach. RBL67 was classified as *Bifidobacterium thermophilum *based on phenotypic and DNA-DNA hybridization characteristics, although 16S rDNA analyses and partial *gro*EL sequences showed higher homology with *B. thermacidophilum *subsp. *porcinum *and *B. thermacidophilum *subsp. *thermacidophilum*, respectively. RBL67 was moderately oxygen-tolerant and was able to grow at pH 4 and at a temperature of 47°C.

**Conclusion:**

In order to assign RBL67 to a species, a polyphasic approach was used. This resulted in the classification of RBL67 as a *Bifidobacterium thermophilum *strain. To our knowledge, this is the first report about *B. thermophilum *isolated from baby faeces since the *B. thermophilum *strains were related to ruminants and swine faeces before. *B. thermophilum *was previously only isolated from animal sources and was therefore suggested to be used as differential species between animal and human contamination. Our findings may disapprove this suggestion and further studies are now conducted to determine whether *B. thermophilum *is distributed broader in human faeces. Furthermore, the postulated differentiation between human and animal strains by growth above 45°C is no longer valid since *B. thermophilum *is able to grow at 47°C. In our study, 16S rDNA and partial *gro*EL sequence analysis were not able to clearly assign RBL67 to a species and were contradictory. Our study suggests that partial *gro*EL sequences may not be reliable as a single tool for species differentiation.

## Background

Since Tissier discovered the *Bifidobacterium *spp. in 1899 [1], over 30 species have been isolated and identified [2] and the first genome sequence of a *Bifidobacterium *is now available [3]. Analyses of amplified partial 16S rDNA sequences assigned to uncultivated bifidobacteria suggest the existence of more new *Bifidobacterium *species[4,5]. Bifidobacteria are known to be heterofermentative anaerobic, Gram-positive bacteria which belong to the class of *Actinobacteria *[6] containing genomes with a high G+C content. They mainly colonize the intestines of humans, other mammals and insects [7]. Some bifidobacteria have also been isolated from environmental sources such as sewage [8]. Bifidobacteria have been described as strictly anaerobic bacteria in the sense that they are not able to grow on agar-plates in the presence of air [7]. However, some *Bifidobacterium *strains were described which were at least partially aerotolerant in the presence of reducing agents in liquid media [9,10]. Genome analyses of *Bifidobacterium longum *suggests that both growth and survival under oxygen pressure are linked with the presence of a set of oxygen-scavenging NADH oxidases [3]. Strains of a novel species, *Bifidobacterium psychraerophilum*, were recently reported to be able to grow under air on the surface of solid agar-medium [11]. Based on heat-shock protein HSP60 encoding sequence homologies, this species clusters distinctly from aerotolerant species of the genera *Scardovia*, *Aeriscardovia*, *Parascardovia *and *Gardnerella *which were related or belonged to the genus *Bifidobacterium *before the recent reclassification [11,12]. For probiotic use of a particular *Bifidobacterium *strain, oxygen tolerance is an important characteristic for maintaining cell viability in end products.

The classification of *Bifidobacterium *species has most often been done in the past mainly by 16S rDNA sequence homology analysis [13] and confirmed by an enzymatic assay of D-fructose-6-phosphate phosphoketolase [14] whose encoding gene *xfp *is widespread among microorganisms and not unique to *Bifidobacterium *species [15]. These results were then substantiated by determinations of DNA-DNA relatedness [16] as well as physiological properties such as carbohydrate fermentation profiles in order to discriminate between single species of the genus *Bifidobacterium*. Recently, phylogenetic trees for bifidobacteria were constructed based on *gro*EL genes encoding heat-shock protein HSP60 [17,18], *gro*ES genes encoding chaperon [18] and *xfp *encoding phosphoketolase [19,20] as alternatives to 16S rDNA-based phylogenetic trees. These trends support the polyphasic approach for species identification as suggested several years ago [21]. The phylogenetic positions of bifidobacteria *gro*EL (synonymly used with HSP60 encoding sequences) seem to generally agree with 16S rDNA-based phylogeny, and in several studies, have been more discriminative than 16S rDNA sequences for species delineation [18]. In a previous study, 34 isolates of *Bifidobacterium *species from infant faeces have been described, six of which showed bacteriocin-like activity against *Listeria monocytogenes *which represents a rare property among bifidobacteria [22]. In this work, the taxonomic position of one of these isolates, strain RBL67, whose properties did not match with any of the so far described *Bifidobacterium *species, was determined using 16S rDNA sequence homology, comparative *gro*EL gene sequence analysis, DNA-DNA genome hybridizations and carbohydrate fermentation patterns. This study aimed to classify RBL67 due to its features which may be used industrially in the future and was not intended to for a complete phylogenetic analysis of *Bifidobacterium *sp.

## Results and discussion

### Phylogenetic position of *Bifidobacterium *sp. RBL67

**Figure 1:**
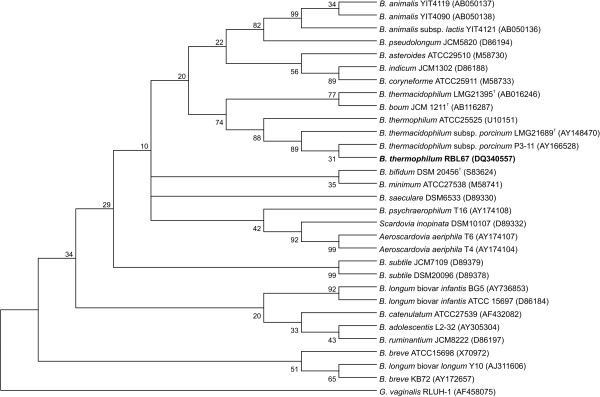
Phylogenetic tree based on 16S rDNA sequences. The tree was rooted with *Gardnerella vaginalis *RLUH-1 and constructed by using the Neighbour-joining method with Jukes-Cantor parameter and a bootstrap value of 1000. The number at each branch point represents percentage bootstrap support. Accession numbers in brackets.

Because 16S rDNA sequences are the most widely used molecules in phylogenetic classification of bacteria, the 16S rDNA fragment of strain RBL67 was amplified by PCR using the lm3/lm26 primer pair (Table 1). The sequence of the resulting fragment (approx. 1.5 kb [Genbank:DQ340557]) showed the highest homologies to 16S rDNA sequences of other bifidobacteria from the GenBank (Table 2) with the highest similarity, 99%, to the sequence of *Bifidobacterium thermacidophilum *subsp. *porcinum *LMG21689^T ^[GenBank: AY148470], isolated by Zhu et al. [17], and 94% to that of the *Bifidobacterium thermophilum *type strain [GenBank: U10151] (Table 2). A phylogenetic tree was constructed from the 16S rDNA sequences using a consensus length of 1392 kb. The tree shows clustering of RBL67 with the *Bifidobacterium thermophilum/thermacidophilum/boum *branch (Fig. 1), described as the "thermophilic group" by Zhu et al. [17].

**Table 1:** Oligonucleotides used in this study

Primer	Sequence (5'-3')
lm3	CGGGTGCTICCCACTTTCATG [42]
lm26	GATTCTGGCTCAGGATGAACG [42]
520F	CAGGAGTGCCAGCAGCCGCGG [25]
520R	ACCGCGGCTGCTGGC [25]
1100F	CAGGAGCAACGAGCGCAACCC [25]
1100R	AGGGTTGCGCTCGTT [25]
H60F	GGNGAYGGNACNACNACNGCNACNG [34]
H60R	TCNCCRAANCCNGGNGCYTTNACNGC [34]
T7	TAATACGACTCACTATAGG [Promega]
SP6	ATTTAGGTGACACTATAG [Promega]

**Table 2:** 16S rDNA sequence homologies of *Bifidobacterium *sp. RBL67 with 16S rDNA sequences found in the GenBank

*Bifidobacterium *sp. RBL67 [GenBank: DQ340557] compared with*	% Identity {Gaps}
*Bifidobacterium thermacidophilum *subsp. *porcinum *LMG 21689^T ^complete sequence [GenBank: AY148470]	99 {2}
*Bifidobacterium boum *JCM1211^T ^16S rDNA partial sequence [Genbank: D86190]	97 {2}
*Bifidobacterium *sp. group I-3 16S ribosomal RNA gene, partial sequence [GenBank: AF321295]	97 {5}
*B. thermacidophilum *subsp. *thermacidophilum *LMG21395^T ^16S rDNA complete sequence [GenBank: AB016246]	96 {12}
*Bifidobacterium saeculare *DSM6533^T ^16S rDNA partial sequence [GenBank: D89330]	95 {6}
*Bifidobacterium subtile *JCM 7109^T ^16S rDNA partial sequence [GenBank: D89329]	95 {6}
*Bifidobacterium thermophilum *ATCC 25525^T ^16S rDNA partial sequence [GenBank: U10151]	94 {8}

*Accession numbers in brackets []

There is insufficient discriminating power to clearly assign a 16S rDNA to a species within the genus *Bifidobacterium *since similarity values for this gene can range from 93% to 99% between species, as reported in other studies [23-25]. Therefore a polyphasic approach, as suggested by Vandamme et al. [21], was used in this study to identify the unknown strain RBL67 at species level.

### Comparative analysis of *gro*EL gene sequences

**Figure 2:**
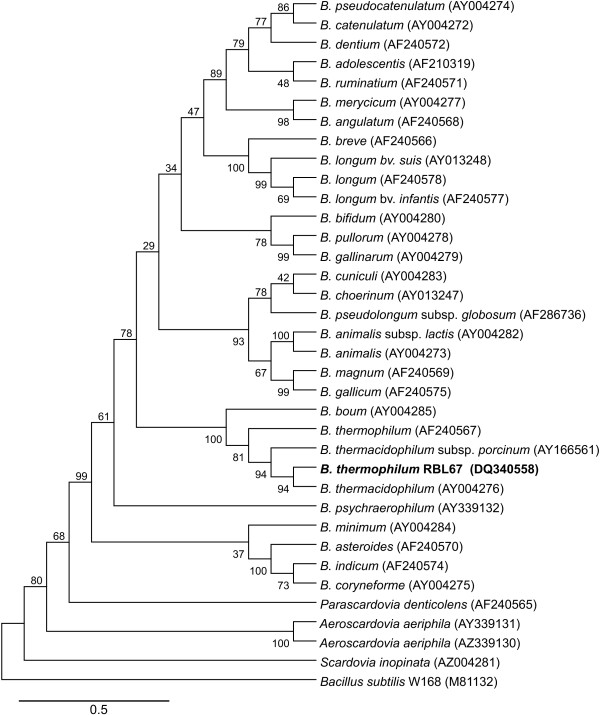
Phylogenetic tree based on fragments of the partial *gro*EL gene DNA sequences rooted with *Bacillus subtilis *W168. The tree was constructed using the neighbour-joining method with Jukes-Cantor parameter and bootstrap values calculated from 1000 trees (represented as percentages at each branch-point). Accession numbers in brackets.

The next step of the classification process was to compare the partial *gro*EL gene sequences (0.59 kb in size) of *Bifidobacterium *RBL67 [GenBank: DQ340558], *B. thermacidophilum *subsp. *thermacidophilum *LMG21395^T ^[GenBank: AY004276] (referred as *B. thermacidophilum *in Fig. 1 and Fig. 2) and *B. thermacidophilum *subsp. *porcinum *LMG21689^T ^[GenBank: AY166561] with those of other bifidobacteria from the GenBank. Based on a consensus length of 0.59 kb, a phylogenetic tree was constructed (Fig. 2) and rooted with *Bacillus subtilis *W168 [GenBank: M81132]. The tree shows common clustering of strain RBL67 with the *Bifidobacterium thermophilum/thermacidophilum/boum *branch, which substantiates the 16S rDNA comparisons presented in Fig. 1. According to the 16S rDNA tree, strain RBL67 splits off earlier on the branch and is closer related to *B. thermophilum *(Fig. 1 [GenBank:U10151 ]) whereas using partial *gro*EL sequences, strain RBL67 is on the same branch as *B. thermacidophilum *(Fig. 2 [GenBank: AY004276]). The comparison of 16S rDNA sequences and partial *gro*EL sequences showed a discrepancy between the classification of strain RBL67 based on those methods. By comparing the partial *gro*EL gene sequences, *Bifidobacterium *RBL67 is more closely related to *B. thermacidophilum *subsp. *thermacidophilum *LMG21395^T ^(98.25% similarity) than to *B. thermacidophilum *subsp. *porcinum *LMG21689^T ^(97.06%, Table 3) whereas based on 16S rDNA sequences strain RBL67 is closer related to *B. thermacidophilum *subsp. *porcinum *LMG21689^T ^(99%, Table 2) than to *B. thermacidophilum *subsp. *thermacidophilum *LMG21395^T ^(96%). According to the definition of Zhu et al. [17] a similarity of 96.5–100% and 95.5–97% is required for intraspecies and inter-subspecies differentiation by partial *gro*EL sequences, respectively. By applying this definition to partial *gro*EL gene sequencing data (Table 3), *Bifidobacterium *RBL67 could belong to the species *thermacidophilum *or it could be a subspecies of *B. thermophilum *DSM20210^T ^(95.65% similarity), which is contradictory. Clearly sequencing analysis of partial *gro*EL could not provide enough evidence to confirm the phylogenetic classification of *Bifidobacterium *isolate RBL67.

**Table 3:** Partial *gro*EL sequence of *Bifidobacterium *sp. RBL67 compared with closely related *Bifidobacterium *species

*Bifidobacterium *sp. RBL67 *gro*EL [GenBank: DQ340558] gene sequence compared with:	% Similarity
*B. thermacidophilum *subsp. *thermacidophilum *LMG 21395^T ^[GenBank: AY004276]	98.25%
*B. thermacidophilum *subsp. *porcinum *LMG 21689^T ^[GenBank: AY166561]	97.06%
*B. thermophilum *DSM 20210^T ^[GenBank: AF240567]	95.65%
*B. boum *DSM 20432^T ^[GenBank: AY004285]	93.58%

### DNA-DNA hybridization and G+C-content

For the final classification of *Bifidobacterium *RBL67, DNA-DNA hybridization was performed. The results (Table 4) confirmed that the partial *gro*EL gene sequence was not sufficient to differentiate between species. According to the limit of 70% similarity required for species identification [26], only *Bifidobacterium boum *DSM20432^T ^could be defined as a different species by DNA-DNA hybridization. *B. thermacidophilum *subsp. *thermacidophilum *LMG21395^T ^was on the limit of this definition; its classification as a new species was justified by data from both DNA-DNA hybridization and phylogenetic data reported by Dong et al. [8]. However, our DNA-DNA hybridizations provide evidence that *B. thermacidophilum *subsp. *porcinum *LMG21689^T ^belongs to *B. thermophilum*, showing 82.25% homology to *B. thermophilum *DSM20210^T ^(Table 4). *Bifidobacterium *RBL67 showed 86.25% homology with *B. thermophilum *DSM20210^T ^and could also not be discriminated as a new species. *Bifidobacterium *RBL67 showed even less similarity to *B. thermacidophilum *subsp. *thermacidophilum *LMG21395^T ^and *B. thermacidophilum *subsp. *porcinum *LMG21689^T ^than to *B. thermophilum *DSM20210^T ^(Table 4). Furthermore the homologies between *Bifidobacterium *RBL67 and both *B. thermacidophilum *strains were similar (77.9% for LMG21395^T ^and 77.2% for LMG21689^T^) while the homology between *B. thermacidophilum *subsp. *thermacidophilum *LMG21395^T ^and *B. thermacidophilum *subsp. *porcinum *LMG21689^T ^was only 71.9%. The result reported by Dong et al. [8] from DNA-DNA hybridization, 58.9% similarity between *B. thermacidophilum *subsp. *thermacidophilum *LMG21395^T ^and *B. thermophilum *DSM20210^T^, was not confirmed in this study. This might be due to different methods of DNA isolation, DNA-DNA hybridization or interpretation of the data.

**Table 4:** Similarity in percent of the DNA-DNA hybridizations of *Bifidobacterium *RBL67, *B. thermacidophilum *subsp. *porcinum *LMG21689^T ^and *B. thermophilum *DSM2010^T ^with closely related strains

	*Bifidobacterium *sp. RBL67	*B. thermacidophilum *subsp. *porcinum *LMG21689^T^	*B. thermophilum *DSM20210^T^
*Bifidobacterium *sp. RBL67	100%	n.d.	n.d.
*B. thermacidophilum *subsp. *thermacidophilum *LMG21395^T^	78%	72%	71%
*B. thermacidophilum *subsp. *porcinum *LMG21689^T^	77%	100%	82%
*B. thermophilum *DSM20210^T^	86%	n. d.	100%
*B. boum *DSM20432^T^	48%	n. d.	n. d.

n. d.: not determined

Based on the data by DNA-DNA hybridization, which is still the strongest method for bacterial species differentiation, we classified strain RBL67 as *Bifidobacterium thermophilum*.

The G+C content of DNA from *Bifidobacterium *RBL67 was 59.7 mol-% as determined by DSMZ Germany. This compared well with the G+C determination of *B. thermophilum *DSM20210^T^, 60 mol-%, but differed from that of *B. thermacidophilum *subsp. *thermacidophilum *LMG21395^T ^and *B. thermacidophilum *subsp. *porcinum *LMG21689^T^, which were 57.7 and 61.5 mol-% G+C, respectively.

### Physiological properties of *Bifidobacterium *sp. RBL67

**Figure 3:**
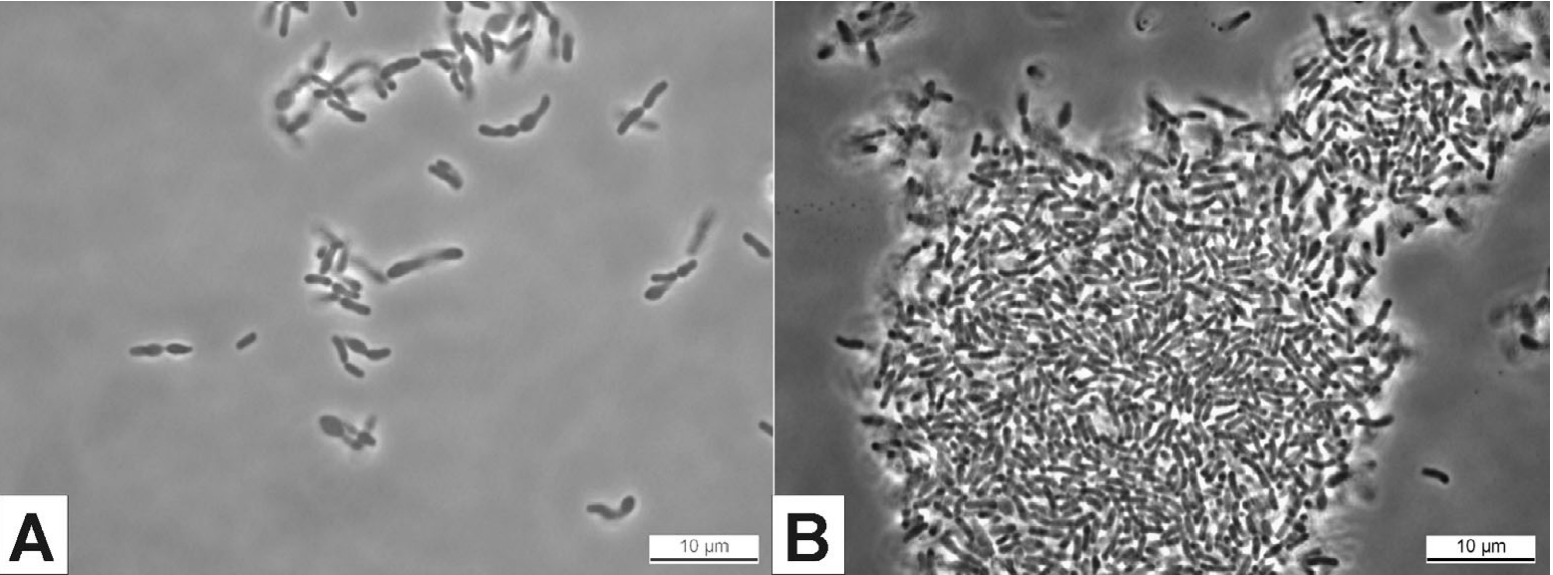
**A: **Microscopic picture of *Bifidobacterium *RBL67 grown on MRS-C agar overnight. White bar indicates 10 μm. **B**: Microscopic picture of a small agglomerated clump of *Bifidobacterium *RBL67 cells in MRS-C liquid culture after 24 h growth at 37°C. White bar indicates 10 μm.

Cells of *Bifidobacterium *RBL67 grew well on MRS-C agar incubated anaerobically at 37°C overnight and on RB-agar at 40°C in 48 h. They were non-motile, irregularly shaped rods and formed pairs when growing on plates (Fig. 3a). On RB-agar, they formed yellow colonies with a diameter of 1 mm. When growing in liquid culture, agglomeration of cells occurred (Fig. 3b). This agglomeration was pH dependent: the lower the pH, the greater the agglomeration of cells. The formation of agglomerates was inhibited by pH control at pH 7 (data not shown). In contrast to *B. thermacidophilum *subsp. *thermacidophilum *LMG21395^T^, aggregated cell clumps of *Bifidobacterium *RBL67 were not easily dispersed again, even after extended mixing.

**Figure 4:**
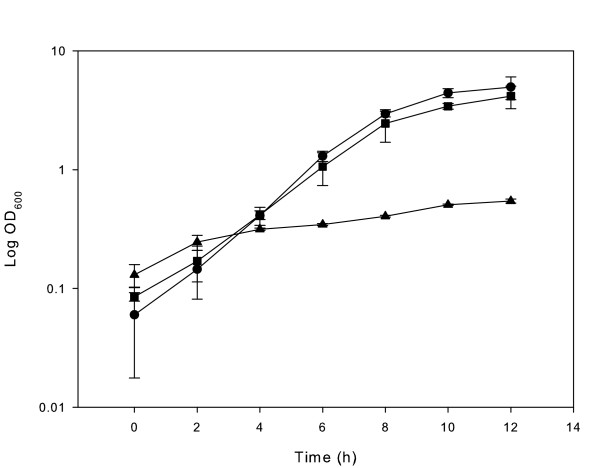
Growth of *Bifidobacterium *RBL67 under different oxygen concentrations in MRS-C at 37°C. Points are mean of three replicates. ● Growth under anaerobic conditions; ■ Growth with 2.5% oxygen; ▲ Growth with 12.5% oxygen.

*Bifidobacterium *RBL67 was moderately oxygen tolerant; therefore it was not necessary to perform dilutions and inoculations anaerobically. However, the strain did not grow on agar plates under aerobic conditions after 7 days. No selected gas atmosphere was necessary to grow the strain in liquid culture. While sparkling the liquid media with oxygen-free nitrogen or carbon dioxide, *Bifidobacterium *RBL67 ceased to grow. To determine the level of oxygen tolerance, anaerobic media were purged with oxygen as described by Meile et al. [10]. Fig. 4 shows the growth curves for *Bifidobacterium *sp. RBL67 at different levels of oxygen in reduced medium. At 2.5% oxygen in the bottle growth of this strain was maintained, showing its elevated tolerance to oxygen. Even at 12.5% oxygen, the OD still increased fivefold during incubation for 12 h. *B. thermacidophilum *subsp. *thermacidophilum *LMG21395^T ^and *B. thermacidophilum *subsp. *porcinum *LMG21689^T ^showed similar growth curves to strain RBL67 under oxidative stress, but seemed to grow slightly better at 12.5% oxygen after 10 h of fermentation (data not shown). Nonetheless, all the strains tested were less oxygen tolerant than *B. animalis *subsp. *lactis*, which was shown to tolerate 50 ml of oxygen in comparable reduced medium [10].

Table 5 shows a summary of the fermentation profiles and growth extremes of *Bifidobacterium *RBL67, RBL68, RBL70, *B. thermacidophilum *subsp. *thermacidophilum *LMG21395^T^, *B. thermacidophilum *subsp. *porcinum *LMG21689^T^, *B. animalis *subsp. *lactis *DSM10140^T ^and *B. thermophilum *DSM20210^T^. For the determination of the growth limits for pH and temperature a limit of OD_600 _≥ 0.4 after 7 days incubation was set. *Bifidobacterium *RBL67 was found to grow in a very large pH and temperature range (Table 5). Its maximum growth temperature (47°C) was very close to that of *B. thermophilum *DSM20210^T^, although the later grew to a higher OD (data not shown). *B. thermacidophilum *subsp. *thermacidophilum *LMG21395^T ^was the only strain in this study that grew at 49°C which confirmed the growth characteristics described by Dong et al. [8].

**Table 5:** Phenotypic characteristics of the tested *Bifidobacterium *species: 67:*Bifidobacterium *sp. RBL67; 68: *Bifidobacterium *sp. RBL68; 70:*Bifidobacterium *sp. RBL70; 21689:*B. thermacidophilum *subsp.*porcinum *LMG21689^T^; 21395: *B. thermacidophilum *subsp.*thermacidophilum *LMG21395^T^; 20210: *B. thermophilum *DSM20210^T^; 10140: *B. animalis *subsp. *lactis *DSM10140^T^

	67	68	70	21689	21395	20210	10140
L-arabinose	-	-	-	-	+	-	+
D-ribose	-	-	-	D	-	-	+
D-galactose	D	+	D	+	+	+	+
D-fructose	+	+	+	+	-	+	D
D-mannose	-	-	-	-	-	-	D
Methyl-AD-mannopyranoside	D	-	-	-	-	D	-
Methyl-AD-glucopyranoside	+	D	-	+	+	+	+
Amygdalin	-	-	-	-	D	+	+
Arbutin	D	-	-	D	D	+	-
Esculin ferric citrate	+	D	-	+	D	+	+
Salicin	-	-	-	D	D	+	+
D-lactose (bovine)	-	-	-	+	-	w	+
D-trehalose	-	-	D	W	-	D	-
Inulin	w	D	w	-	-	D	-
Gentiobiose	-	-	-	-	-	+	D
D-melezitose	-	-	D	-	-	D	-
Minimum growth temp. (°C)	≤25	≤25	n. d.	≤25	≤25	≤25	≤25
Maximum growth temp. (°C)	47	47	47	47	49	47	46
Minimum growth pH	≤4	≤4.5	≤4	≤4.5	≤4.5	≤4.2	≤4.5
DNA G+C content (mol-%)	59.7	n. d.	n. d.	61.5	57.7	60	61.9

+, positive reaction; -, negative reaction; w, weak reaction; D, variable reaction → colour could not be assigned to either yellow or blue; n. d.: not determined

As for a lower pH limit, RBL67 and RBL70 were the only strains still growing at pH 4 under the growth conditions of the study. *B. thermacidophilum *subsp. *thermacidophilum *LMG21395^T ^did not grow at this pH, which was reported by Dong et al. [8]. The upper pH limit for growth was not determined, but all tested cultures still grew very well at pH 8. The molar ratio of acetate to lactate from glucose for *Bifidobacterium *RBL67 was determined as 5.91 to 1 in MRS-C medium at 37°C under anaerobic conditions.

## Conclusion

In order to classify the *Bifidobacterium *isolate RBL67 at species level, we used a polyphasic approach since phylogenetic trees based on partial *gro*EL gene (as used by Ventura et al. [18] and Dong et al. [8]) and 16S rDNA sequences generated similar but not identical results for species classification. Berthoud et al. [20] reported similar difficulties when studying the "thermophilic group", which could not be differentiated at the species level by *xfp *gene sequencing. They proposed the use of 16S rDNA sequencing, as an additional method for species differentiation. However, our DNA-DNA hybridization data (Table 4) revealed a very close relationship between *B. thermophilum *species and *B. thermacidophilum *species and questioned the current classification of *B. thermacidophilum *as a discrete species. Our hybridization data were substantiated by phylogenetic data, molecular characterization and physiological properties and allowed the classification of strain RBL67 into the species *Bifidobacterium thermophilum*. In contrast with a previous report [8], among our tested strains (Table 4) only *B. boum *could be clearly classified as a species different from *B. thermophilum *based on DNA-DNA hybridization. This finding suggests that partial *gro*EL gene sequences may not be reliable as a single tool for *Bifidobacterium *species differentiation. However, more studies have to be done to confirm this suggestion.

As stated before, we did not intend to perform a complete phylogenetic analysis. However, as more data (16S rDNA sequences, *gro*EL sequences) become available on strains closely related to *B. thermophilum *RBL67, a complete phylogenetic analysis could be performed which may lead to a new definition of the *boum/thermophilum/thermacidophilum *branch.

A very important finding of this study is that *B. thermophilum *could be isolated from human origin (baby faeces). In previous studies, *B. thermophilum *has been classified as an animal-related species mainly present in ruminant faeces [27,28]. Since it was previously possible to differentiate between animal and human bifidobacteria by species identification, it has been suggested that bifidobacteria should be used to discriminate between animal and human bacterial contamination in foods [27,29]. Because *B. thermophilum *RBL67 was isolated from baby faeces, it is likely that this species can not be used as animal contamination indicator. On the other hand, Gavini et al. [30] detected *B. adolescentis*, a species predominantly found in human, in cow dung, swine and rabbit faeces which restricts the number of bifidobacterial species that can be used for human faecal contamination detection.

Another discrimination tool between human and animal bifidobacteria strains was the temperature growth limit of 45°C for strains from human origin [7,31]. *B. thermophilum *strains are able to grow at 47°C and since they can also be found in human faeces [this study], the use of the temperature is not reliable anymore for the discrimination of the origin of bifidobacteria. However, since only a few species are able to grow above 45°C, the assignment to a *Bifidobacterium *sp. can be narrowed. Further studies are now undergoing to determine whether *B. thermophilum *is more widespread within humans.

## Methods

### Bacterial strains and routine growth conditions

A list of strains used in this study is presented in Table 6. Strains were kept in their respective growth media (see below) supplemented with 30% (v/v) glycerol at -80°C. Prior to use, all bifidobacteria were grown on Raffinose-Bifidobacterium (RB)-agar plates [32], without sodium caseinate, supplemented with 1.5% (v/v) agar (Oxoid). After 5 sub cultivations they were transferred to MRS-C broth consisting of MRS with 0.1% (v/v) Tween 80 (Biolife) and 0.05% (w/v) L-cysteine-hydrochloride (Sigma) or on MRS-C agar (MRS-C broth with 1.5% (w/v) agar). *Bifidobacterium bifidum *DSM20456^T ^was cultivated only on MRS-C agar as it didn't grow on RB-agar. All strains were incubated anaerobically at 37°C overnight in MRS-C broth and agar, or at 40°C for 48 h on RB-agar.

**Table 6:** Strains used in this study

Species	Strain
*Bifidobacterium thermacidophilum *subsp. *thermacidophilum*	LMG 21395^T^
*Bifidobacterium thermacidophilum *subsp. *porcinum*	LMG 21689^T^
*Bifidobacterium adolescentis*	DSM 20083^T^
*Bifidobacterium bifidum*	DSM 20456^T^
*Bifidobacterium boum*	DSM 20432^T^
*Bifidobacterium breve*	DSM 20213^T^
*Bifidobacterium longum *subtype *infantis*	DSM 20088^T^
*Bifidobacterium longum *subtype *longum*	DSM 20219^T^
*Bifidobacterium thermophilum*	DSM 20210^T^
*Bifidobacterium longum *subtype *suis*	DSM 20211
*Bifidobacterium animalis *subsp. *Lactis*	DSM 10140^T^
*Bifidobacterium*	RBL67*
*Bifidobacterium*	RBL68*
*Bifidobacterium*	RBL70*
*Escherichia coli*	XL1-Blue‡

BCCM/LMG™: Belgian co-ordinated collections of micro-organisms/Laboratorium voor

Microbiology en microbiele Genetica, Ghent, Belgium

DSM: German Microorganism Collection, Braunschweig, Germany

*Isolated from baby faeces [22]; ‡ referred in Bullock et al. [41]

### Growth under oxidative-, heat- and pH-stress conditions

Growth under conditions of oxidative stress was measured after a modified method described by Meile et al. [10]. Volumes of 0, 10 and 50 ml of pure oxygen were added to sterile serum flasks containing 400 ml of MRS-C. The flasks were inoculated with 1% of an overnight culture of either *B. longum *subtype *longum *DSM 20219^T^, *Bifidobacterium *RBL67, *B. thermacidophilum *subsp. *thermacidophilum *LMG 21395^T ^or *B. thermacidophilum *subsp. *porcinum *LMG 21689^T^. The flasks were then incubated in a shaker at 160 rpm and 37°C for 12 h. Samples were taken every 2 h and treated for 3 min in a stomacher to remove clumps prior to measurements of the optical density at 600 nm. Each growth curve was carried out twice.

The temperature and pH extremes of *Bifidobacterium *RBL67, RBL68 and RBL70, *B. thermacidophilum *subsp. *thermacidophilum *LMG 21395^T^, *B. thermacidophilum *subsp. *porcinum *LMG 21689^T^, *B. animalis *subsp. *lactis *DSM 10140^T ^and *B. thermophilum *DSM 20210^T ^were determined as follows:

25 ml of MRS-C containing 2 mg l^-1 ^resazurin as redox indicator were anaerobically inoculated with 1% of the tested bifidobacteria cultured overnight. The temperature range was evaluated by incubating the strains for 7 days at 12, 25, 46, 47, 48 and 49°C with an initial pH of 7.0. The pH range was determined using pH 4.0, 4.5, 5.0, 5.5 and 8.0 as initial pH for growth. Samples were incubated at 37°C for 7 days. Aliquots were taken daily, treated in a stomacher for 3 min and the OD was measured at 600 nm. Each growth condition was measured twice. If a strain failed to reach OD_600 _of 0.4 after 7 days, it was declared as not growing under the tested conditions.

### Amplification and sequencing of 16S rDNA

Specific amplification of the 16S rDNA of *Bifidobacterium *RBL67 and other bifidobacteria was done using a slightly modified PCR protocol established by Schürch [33] using the primer pair lm3/lm26 (Table 1). The annealing temperature used was 62°C instead of 60°C. After agarose gel electrophoresis the corresponding band for 16S rDNA at the 1.5 kb position was cut out and purified using the GFX PCR purification kit (Amersham Biosciences). Sequencing of the PCR product was done by Microsynth GmbH using the primers 520F, 520R, 1100F and 1100R (Table 1). Sequence analysis and comparison was done using the GCG software package version 10 as described before [34]. Phylogenetic and molecular evolutionary analyses were conducted using *MEGA *version 3.1 [35]. The sequences were aligned with ClustalW (version 1.6) and the tree was calculated using the neighbour-joining method with Jukes-Cantor parameter and a bootstrap value of 1000.

### Cloning and analysis of *gro*EL sequences

DNA cloning and sequencing of a partial heat shock *gro*EL gene sequence from *Bifidobacterium *RBL67 were done using a modified method of Jian et al. [34]. The DNA template was extracted using the method of Leenhouts et al. [36]. To amplify part of a gene fragment with PCR, the following reaction mixture (25 μl) was used: 3–30 ng DNA template measured at 260 nm with a Eppendorf-Biophotometer, 2.5 U Taq polymerase (Euroclone), 0.1 mM dNTP's (Amersham Biosciences), 1.5 mM MgCl_2_, 2 μM each of primer H60R and H60F (Table 1) and 2.5 μl 10 × PCR buffer (Euroclone). The PCR reactions were carried out in a Biometra Tgradient Thermal Cycler using the following protocol: denaturation step 95°C for 5 min followed by 40 cycles of 94°C for 30 s, 50°C for 30 s and 72°C for 1 min at a heating rate of 1.5°C min^-1^. At the end, the temperature was maintained at 72°C for 10 min. A 25-μl aliquot of the reaction mixtures was mixed with 10 μl Orange G loading dye (0.25% Orange G from Fluka in 30% glycerol) and subjected to electrophoresis on agarose (0.8%) gels in 1 × TAE buffer. DNA bands were visualized by 2.5 μg ml^-1 ^ethidium bromide under UV light. Corresponding bands (0.59 kb in size) were then cut out and purified as described above.

Purified PCR fragments were ligated into the pGEM-T easy vector using the Promega PCR cloning kit. Ligation and cloning was performed according to the kit manual. Transformation of *E. coli *XL-1 blue cells was done using the electroporation method described by Sambrook and Russel [37]. Recombinant plasmids were extracted from transformed cells with the Promega Wizard Plus Midiprep DNA Purification System. Plasmid DNA (100 ng μl^-1^) carrying partial HSP60 encoding gene sequences from *Bifidobacterium *RBL67 or *B. thermacidophilum *subsp. *thermacidophilum *LMG 21395^T ^was then sequenced by Microsynth GmbH and finally aligned to the corresponding sequences from bifidobacteria and *Bacillus subtilis *W168 (obtained from Genbank entries) using the CLUSTAL W software (version 1.6). Similarities were calculated and converted into a distance matrix with the Jukes-Cantor parameter and rooted with *Bacillus subtilis *W168 applying a bootstrap value of 1000 using the software *MEGA *version 3.1 [35].

### DNA-DNA hybridization

Whole genome DNA-DNA hybridizations were carried out externally at DSMZ Germany. Hybridizations were performed with the genomes of *Bifidobacterium *RBL67, *B. thermacidophilum *subsp. *thermacidophilum *LMG21395^T^, *B. thermacidophilum *subsp. *porcinum *LMG21689^T^, *B. thermophilum *DSM 20210^T ^and *B. boum *20432^T^. Total DNA was isolated using a French pressure cell according to the method described by Cashion et al. [38], DNA-DNA hybridizations were carried out according to De Ley et al. [39] with modifications of Huss et al. [40] in 2 × SSC and 10% formamide (v/v) at a temperature of 67°C. The analyses were performed with a model Cary 100 Bio UV/VIS-spectrophotometer equipped with a Peltier-thermostated 6 × 6 multicell changer and a temperature controller with *in situ *temperature probe.

### Carbohydrate fermentation and acid analysis

Carbohydrate fermentation of strain RBL67 was analyzed using API 50 CHL strips (Biomérieux). The tests were carried out in triplicates according to the manufacturer's instructions with a modification in culture preparation. Briefly, 2 ml of an overnight culture in MRS-C broth were centrifuged (14000 g, 5 min, 4°C). The pellet was then washed and resuspended in 1 ml of autoclaved water. This suspension was then mixed with 5 ml CHL50 medium (Biomérieux) and 0.1 ml of this mixture was applied to each tube of the API test. The test strips were then incubated anaerobically for 72 h at 37°C and evaluated after 24, 48 and 72 h.

Molar ratio of acetic and lactic acid was determined using HPLC. 2 ml of an overnight culture (16 h) of either *Bifidobacterium *RBL67, RBL68 or RBL70, *B. thermacidophilum *subsp. *thermacidophilum *LMG 21395^T ^or *B. thermacidophilum *subsp. *porcinum *LMG 21689^T ^were centrifuged (14000 g, 5 min, 4°C). The supernatant was 10 × diluted in HPLC-grade water and filtered (0.45 μm) prior to HPLC analysis. This was carried out using an Aminex HPX-87H (300 × 7.8 mm) column (Bio-Rad) in a Merck LaChrom HPLC system. Sulfuric acid (10 mM, Fluka) was used as mobile phase at a flow rate of 0.4 ml min^-1^. Sugars and acids were detected by a RI detector. Analyses were done in duplicate.

## Authors' contributions

UVA did the first identification and characterization experiments, carried out the sequence alignments, prepared the DNA-DNA hybridizations, coordinated the study and drafted the manuscripts. VM did the molecular and phenotypic experiments, statistical analysis and participated in sequence alignment and DNA-DNA hybridization preparation. CL conceived the study and participated in the coordination of the study. LM participated in the coordination of the study and the sequence interpretation and helped in the draft of the manuscript. IF and EEK were responsible for the screening and isolation of bifidobacteria strains from baby faeces. All authors read and approved the final manuscript.

## References

[B1] Tissier MH (1899). La réaction chromophile d'Escherich et Bacterium Coli. C R Seances Soc Biol Fil.

[B2] Klijn A, Mercenier A, Arigoni F (2005). Lessons from the genomes of bifidobacteria. FEMS Microbiol Rev.

[B3] Schell MA, Karmirantzou M, Snel B, Vilanova D, Berger B, Pessi G, Zwahlen M-C, Desiere F, Bork P, Delley M (2002). The genome sequence of *Bifidobacterium longum *reflects its adaptation to the human gastrointestinal tract. Proc Natl Acad Sci USA.

[B4] Ley RE, Peterson DA, Gordon JI (2006). Ecological and Evolutionary Forces Shaping Microbial Diversity in the Human Intestine. Cell.

[B5] Satokari RM, Vaughan EE, Smidt H, Saarela M, Matto J, de Vos M (2003). Molecular approaches for the detection and identification of bifidobacteria and lactobacilli in the human gastrointestinal tract. System Appl Microbiol.

[B6] Stackebrandt E, Rainey FA, Ward-Rainey NL (1997). Proposal for a new hierarchic classification system, *Actinobacteria classis nov*. Int J Syst Bacteriol.

[B7] Biavati B, Mattarelli P, Dworkin M, Falkow S, Rosenberg E, Schleifer KH, Stackebrandt E (2005). The family *Bifidobacteriaceae*. The prokaryotes: an evolving electronic resource for the microbiological community.

[B8] Dong X, Xin Y, Jian W, Liu X, Ling D (2000). *Bifidobacterium thermacidophilum sp. nov*., isolated from an anaerobic digester. Int J Syst Bacteriol.

[B9] Beerens H, Gavini F, Neut C (2000). Effect of exposure to air on 84 strains of bifidobacteria. Anaerobe.

[B10] Meile L, Ludwig W, Rueger U, Gut C, Kaufmann P, Dasen G, Wenger S, Teuber M (1997). *Bifidobacterium lactis *sp. *nov*, a moderately oxygen tolerant species isolated from fermented milk. System Appl Microbiol.

[B11] Simpson PJ, Ross RP, Fitzgerald GF, Stanton C (2004). *Bifidobacterium psychraerophilum *sp. *nov*. and *Aeriscardovia aeriphila *gen. *nov*., sp. *nov*., isolated from a porcine caecum. Int J Syst Bacteriol.

[B12] Jian W, Dong X (2002). Transfer of *Bifidobacterium inopinatum *and *Bifidobacterium denticolens *to *Scardovia inopinata *gen. *nov., comb. nov*., and *Parascardovia denticolens *gen. *nov., comb. nov*., respectively. Int J Syst Bacteriol.

[B13] Leblond-Bourget N, Philippe H, Mangin I, Decaris B (1996). 16S rRNA and 16S to 23S internal transcribed spacer sequence analyses reveal inter- and intraspecific *Bifidobacterium *phylogeny. Int J Syst Bacteriol.

[B14] Scardovi V, Trovatelli LD (1965). The fructose-6-phosphate shunt as peculiar pattern of hexose degradation in the genus *Bifidobacterium*. Ann Microbiol Enzim.

[B15] Meile L, Rohr LM, Geissmann TA, Herensperger M, Teuber M (2001). Characterization of the D-xylulose 5-phosphate/D-fructose 6-phosphate phosphoketolase gene (*xfp*) from *Bifidobacterium lactis*. J Bacteriol.

[B16] Stackebrandt E, Goebel BM (1994). Taxonomic note: a place for DNA-DNA reassociation and 16S rRNA sequence analysis in the present species definition in bacteriology. Int J Syst Bacteriol.

[B17] Zhu L, Li W, Dong X (2003). Species identification of genus *Bifidobacterium *based on partial HSP60 gene sequences and proposal of *Bifidobacterium thermacidophilum *subsp. *porcinum *subsp. *nov*. Int J Syst Bacteriol.

[B18] Ventura M, Canchaya C, Zink R, Fitzgerald GF, van Sinderen D (2004). Characterization of the *gro *EL and *gro*ES loci in *Bifidobacterium breve *UCC 2003: genetic, transcriptional, and phylogenetic analyses. Appl Environ Microbiol.

[B19] Yin X, Chambers JR, Barlow K, Park AS, Wheatcroft R (2005). The gene encoding xylulose-5-phosphate/fructose-6-phosphate phosphoketolase (*xfp*) is conserved among *Bifidobacterium *species within a more variable region of the genome and both are useful for strain identification. FEMS Microbiol Lett.

[B20] Berthoud H, Chavagnat F, Haueter M, Casey MG (2005). Comparison of partial gene sequences encoding a phosphoketolase for the identification of bifidobacteria. LMWT.

[B21] Vandamme P, Pot B, Gillis M, Vos Pd, Kersters K, Swings J (1996). Polyphasic taxonomy, a consensus approach to bacterial systematics. Microbiol Rev.

[B22] Touré R, Kheadr E, Lacroix C, Moroni O, Fliss I (2003). Production of antibacterial substances by bifidobacterial isolates from infant stool active against *Listeria monocytogenes *. J Appl Microbiol.

[B23] Satokari R (2002). Molecular identification and characterisation of bifidobacteria and lactobacilli in the human gastrointestinal tract. PhD thesis.

[B24] Vaugien L, Prevots F, Roques C (2002). Bifidobacteria identification based on 16S rRNA and pyruvate kinase partial gene sequence analysis. Anaerobe.

[B25] Miyake T, Watanabe K, Watanabe T, Oyaizu H (1998). Phylogenetic analysis of the genus *Bifidobacterium *and related genera based on 16S rDNA sequences. Microbiol Immunol.

[B26] Wayne LG, Brenner DJ, Colwell RR, Grimont PAD, Kandler O, Krichevsky MI, Moore LH, Moore WEC, Murray RGE, Stackebrandt E (1987). Report of the ad hoc committee on reconciliation of approaches to bacterial systematics. Int J Syst Bacteriol.

[B27] Delcenserie V, Bechoux N, Leonard T, China B, Daube G (2004). Discrimination between *Bifidobacterium *species from human and animal origin by PCR-restriction fragment length polymorphism. J Food Prot.

[B28] Klein G, Pack A, Bonaparte C, Reuter G (1998). Taxonomy and physiology of probiotic lactic acid bacteria. Int J Food Microbiol.

[B29] Beerens H (1998). Bifidobacteria as indicators of faecal contamination in meat and meat products: detection, determination of origin and comparison with *Escherichia coli *. Int J Food Microbiol.

[B30] Gavini F, Delcenserie V, Kopeinig K, Pollinger S, Beerens H, Bonaparte C, Upmann M (2006). *Bifidobacterium *species isolated from animal feces and from beef and pork meat. J Food Prot.

[B31] Gavini F, Pourcher AM, Neut C, Monget D, Romond C, Oger C, Izard D (1991). Phenotypic differentiation of bifidobacteria of human and animal origins. Int J Syst Bacteriol.

[B32] Hartemink R, Kok BJ, Weenk GH, Rombouts FM (1996). Raffinose-Bifidobacterium (RB) agar, a new selective medium for bifidobacteria. J Microbiol Meth.

[B33] Schürch C (2002). Development of a novel DNA transformation system for bifidobacteria. PhD thesis.

[B34] Jian W, Zhu L, Dong X (2001). New approach to phylogenetic analysis of the genus *Bifidobacterium *based on partial HSP60 gene sequences. Int J Syst Bacteriol.

[B35] Kumar S, Tamura K, Nei M (2004). MEGA3: Integrated software for Molecular Evolutionary Genetics Analysis and sequence alignment. Brief Bioinform.

[B36] Leenhouts KJ, Kok J, Venema G (1989). Campbell-like integration of heterologous plasmid DNA into the chromosome of *Lactococcus lactis *subsp. *lactis*. Appl Environ Microbiol.

[B37] Sambrook J, Russel DW (2001). Molecular Cloning: a laboratory manual.

[B38] Cashion P, Hodler-Franklin MA, McCully J, Franklin M (1977). A rapid method for base ratio determination of bacterial DNA. Anal Biochem.

[B39] Ley JD, Cattoir H, Reynaerts A (1970). The quantitative measurement of DNA hybridization from renaturation rates. Eur J Biochem.

[B40] Huss VAR, Festl H, Schleifer KH (1983). Studies on the spectrophotometric determination of DNA hybridization from renaturation rates. System Appl Microbiol.

[B41] Bullock WO, Fernandez JM, Short JM (1987). XL1-Blue: a high efficiency plasmid transforming *rec*A *Escherichia coli *strain with beta-galactoside selection. BioTechn.

[B42] Kaufmann P, Pfefferkorn A, Teuber M, Meile L (1997). Identification and quantification of *Bifidobacterium *species isolated from food with genus-specific 16S rRNA-targeted probes by colony hybridization and PCR. Appl Environ Microbiol.

